# Gray Matter Volume of the Lingual Gyrus Mediates the Relationship between Inhibition Function and Divergent Thinking

**DOI:** 10.3389/fpsyg.2016.01532

**Published:** 2016-10-03

**Authors:** Lijie Zhang, Lei Qiao, Qunlin Chen, Wenjing Yang, Mengsi Xu, Xiaonan Yao, Jiang Qiu, Dong Yang

**Affiliations:** ^1^School of Psychology, Southwest UniversityChongqing, China; ^2^Key Laboratory of Cognition and Personality, Ministry of Education, Southwest UniversityChongqing, China

**Keywords:** creativity, divergent thinking, fluency, gray matter volume, inhibition, lingual gyrus, long-term memory, originality

## Abstract

Although previous research provides converging evidence for the role of posterior regions of the brain (including temporal, occipital, and parietal regions) involved in inhibition on creative thinking, it remains unclear as to how these regions influence individual differences in creative thinking. Thus, we explored the relationship between posterior regions (i.e., hippocampal, parahippocampal, lingual gyrus, precuneus, and cuneus), inhibition function, and divergent thinking (DT) in 128 healthy college students. The results revealed that lower inhibition was associated with larger gray matter volume (GMV) in the lingual gyrus, which in turn was associated with higher DT. In addition, GMV in the lingual gyrus mediated the association between inhibition and DT. These results provide new evidence for the role of inhibition in creative thinking. Inhibition may affect the amount of information stored in long-term memory, which, in turn influences DT.

## Introduction

Creativity can be defined as the production of a novel, useful, and surprising solution to a complex problem (Sternberg and Lubart, 1996) and is crucial to several aspects of human life (Simonton, 2000). DT, the essence of creative thinking (Guildford, 1987; Zhu et al., 2013), is a complex process that is regulated by multiple cognitive and neural factors (Ward et al., 1999; Benedek et al., 2014; Boccia et al., 2015b). Alpha synchronization in creative ideation was linked to prefrontal and right parietal and occipital sites. Increases in prefrontal alpha activity are usually observed in cognitive tasks involving high top-down control (e.g., inhibition function). In contrast, alpha synchronization at posterior parietal and occipital sites appears to be more specific to cognitive processes involved in creativity (Fink and Benedek, 2014). Further, Dietrich (2004) described that task-relevant memory stored in the TOP areas is pulled by the prefrontal cortex which acts as a search engine and then temporarily represent it in working memory. The prefrontal cortex’s cognitive flexibility allows for retrieved information to be combined. The functions of posterior regions, including TOP areas, differ from those of the prefrontal cortex (Dietrich, 2004). The TOP nerve cells are applied mainly to sensory feelings and long-term memory. However, the mechanism of creativity-related cognition remains unclear. Therefore, we adopted an inhibition-TOP-DT approach to investigate the links between inhibition, posterior brain regions, and DT.

Recent investigations have consistently linked inhibition function to DT. For instance, creative people with fluency of ideas and associations (Mednick et al., 1964; Benedek et al., 2012b), are characterized by a lack of inhibition function to filter task-irrelevant information (Eysenck, 1995; Martindale, 1999), and appear to show over-inclusive thinking (Carson et al., 2003). Moreover, the frequency and originality of ideas is facilitated when resources for inhibition are lacking (Radel et al., 2015). Studies in the field of psychology and psychiatry show that several mental states associated with impaired executive functioning can lead to creative performance (Dietrich, 2004; Healey and Rucklidge, 2006; White and Shah, 2006; Keri, 2009; Morgan et al., 2010; Wieth and Zacks, 2011; Chrysikou et al., 2013; Radel et al., 2015). However, contrary positions on the relationship between the creativity and inhibition exist (Golden, 1975; Groborz and Necka, 2003; Benedek et al., 2014; Edl et al., 2014). For instance, a positive correlation between inhibition – measured by random motor generation tasks – and ideational fluency was observed (Benedek et al., 2012a; Zabelina et al., 2012). Obviously, the contradiction exists across abovementioned studies. Therefore, some researchers in the creative field speculated that creative cognition may involve a two-stage process including idea generation and idea evaluation (Finke et al., 1992). These researchers commonly think idea generation as a bottom-up process which is associated with diffuse attention. By contrast, idea evaluation is regarded as a top-down process with focused attention and cognitive control (Jung et al., 2013). Furthermore, it has been suggested by a model that idea generation and idea evaluation are separately depend on the default and the control networks (Jung et al., 2013). A series of studies suggest that creative thought involves cooperation between idea generation and idea evaluation or cooperation between the default and the control networks (Dietrich and Kanso, 2010; Ellamil et al., 2012; Benedek et al., 2013; Beaty et al., 2014b, 2016). For example, it is indicated that both effects of semantic distance (related with ideas generation) and executive function (related with ideas evaluation) variables predict the DT in the same structural equation model (Beaty et al., 2014b).

On the one hand, functional imaging studies have repeatedly demonstrated inhibition control cortex plays a crucial role in the creative thinking (Fink et al., 2009; Abraham et al., 2012a,b; Kleibeuker et al., 2013a,b; Beaty et al., 2014a; Pinho et al., 2014; Zhang et al., 2014; Fink et al., 2015; Mayseless et al., 2015). Importantly, inferior frontal gyrus (IFG) is classically associated with inhibitory control (Aron et al., 2004, 2014; Houdé et al., 2010). On the other hand, structural and neuroscience evidence indicated the role of TOP in promoting DT. TOP regions are the primary sensory cortices of all sense modalities. Sensory information initially decoded in the primary cortex is further assembled and assimilated the TOP regions responsible for association. It is widely accepted that TOP regions are the sites of long-term memory storage (Gilbert, 2001; Dietrich, 2004). In addition, hippocampal and parahippocampal regions are involved in verbal DT (Fink et al., 2009). Furthermore, the middle occipital gyrus and parietal lobes which are drawn into mental imagery, such as (Sack et al., 2005; Belardinelli et al., 2009; Boccia et al., 2015a), may be involved during creative thinking. Jauk et al. (2015) discovered that cuneus/lingual gyrus were structural substrates of ideational fluency. Jung et al. (2010) revealed that cortical thickness of the lingual gyrus and cuneus was negatively correlated with DT. Zhang et al. (2014) also discovered that the left lingual gyrus (BA 18) underlying relevant visual imagery is involved in the process of semantic relatedness. They inferred that the lingual gyrus may take part in visual imagery in this process. According to Fink et al. (2014), enhanced gray matter density in the right precuneus and the right cuneus may be indicative of vivid imaginative abilities in more creative individuals. The volumetric approach is preferable in studying inhibition function, as structural changes may reveal more stable individual differences relative to task-related activations identified in functional neuroimaging research (DeYoung et al., 2010).

Though little empirical evidence suggests a direct link between inhibition function and TOP regions, it is noteworthy that the inhibition function – a component of executive function – can filter task-irrelevant information that can therefore disrupt goal completion (Dempster, 1992; Miyake et al., 2000; Radel et al., 2015). In other words, a lack of inhibition function result in a broader range of information penetrating working memory space (Radel et al., 2015). A broader range of information in working memory space contributes to more information stored in long-term memory (Norman, 2013). More importantly, Dietrich (2004) proposed that the frontal lobe, which is responsible for executive functions, allows cognitive freedom and flexibility, thereby freeing us from the confine of direct environmental information or memory stored in TOP regions; this is at the core of creativity theories such as Guilford’s (1967) concept of DT.

Collectively, the literature indicates a three-way relationship between inhibition function, TOP, and DT, although the direction of influences is unclear. Therefore, the mediation hypothesis in the present study states that individuals with poorer inhibition function would perform better at DT tasks. Poorer inhibition function may increase the amount information stored in brain regions associated with long-term memory, and the demand for large information storage contributes to increased volumes in these brain regions. In addition, evidence suggests that brain structures represent perceptual- and cognitive-level variables to some degree (Kanai and Rees, 2011; Montag et al., 2013; Gilaie-Dotan et al., 2014). More information helps form new combinations. Based on previous evidence linking inhibition function and the three posterior cortices (TOP) to DT (Boccia et al., 2015b; Liu et al., 2015; Lustenberger et al., 2015) and inhibition function to DT (Radel et al., 2015), the present study investigated the associations among GMV of classical regions located in TOP, inhibition function, and DT. In addition, we examined the potential mediating role of GMV in these ROIs in the relationship between inhibition function and DT. Furthermore, IFG-a classical brain region associated with inhibition function-is related to originality and appropriateness of the ideas proposed by the participants (Aron et al., 2004, 2014; Houdé et al., 2010). We expected that (a) the volume of ROIs located in TOP would be positively associated with DT and negatively associated with inhibition function. Furthermore, inhibition function would be negatively associated with DT; (b) the volume of ROIs located in TOP would mediate the relationship between the inhibition and DT; (c) the volume of IFG is related with inhibition function and DT. The multidimensional approach employed in the present study has the potential to advance our understanding of the cognitive and neural factors influencing DT.

## Materials and Methods

### Subjects

Participants comprised 128 right-handed, healthy subjects (49 males; age: Mean = 19.69 years; range = 17–22) who took part in our project to investigate the relationships among creativity, mental health, and brain imaging (Li et al., 2014; Wei et al., 2014). All of them were undergraduates from the Southwest University, China. Based on a self-report questionnaire administered before the scan, we found that none experienced neurological or psychiatric illness, and substance abuse. All participants signed the written informed consent. The study was granted by the Institutional Review Board of Southwest University Imaging Center for Brain Research.

### Assessing Inhibition

The Stroop color–word-interference task was used to assess the inhibition of prepotent responses (Stroop, 1935). It is generally thought to be a classical inhibition task (Miyake et al., 2000). The task involves presenting four words (“red”, “green”, “blue,” or “yellow”) which denote any a color name in the middle of a black computer screen. All words are segmented into congruent trials or in congruent trials. Congruent trails signify that the color of each word was consistent with its meaning. On the contrary, incongruent trails imply that the color of each word was inconsistent with its meaning. In this study, the task included one practice block, and four task blocks, each comprising 24 trials: 12 incongruent, and 12 congruent trials. Incongruent and congruent trails are presented in a random order within each block. Participants were requested to identify the color of word as quickly as possible within 4 s by pressing one of four appointed keys (D, F, J, K). After an incorrect response feedback will be presented to participants to ensure high accuracy. In the presence of incongruent trials participants require suppressing the dominant process of word naming. Mean response time difference between incongruent trails and congruent trails in each block is taken as the interference score. However, the interference score in the Stroop task is reverse to inhibitory control. Thus, the negative interference score was used to the next step analysis.

### Assessing Creativity

The Torrance (1974) Tests of Creative Thinking – based on DT – is the most commonly used test (Cramond et al., 2005; Krumm et al., 2014a,b). We used three modified product-improvement tasks, which are a kind of verbal DT tasks from Torrance’s tests. This task requires participants to improve a product, particularly a toy, such that they would enjoy the activity. It differs considerably from extremely popular, Alternate uses test (Christensen et al., 1960). Differing from traditional verbal evaluations of the Torrance Tests of Creative Thinking, we only focus on fluency and originality of DT: they reflect quantity of ideas in a limited time and quality, respectively (Jauk et al., 2014).

Participants were given 3 min per task to write all the novel and appropriate responses that they could think of on a personal computer. A consensual assessment technique was used to assess the ideas (Amabile et al., 1990). All responses were assessed for originality and fluency by four experienced professionals using a similar 10-point scale. Originality means the degree of originality of the responses (i.e., the quality of ideas) and fluency reflects the number of ideas produced by a participant (i.e., quantity of ideas). For each task, originality and fluency scores reflected the average originality and fluency rating of the three ideas assessed by the raters. In the next step analysis, we analysis the two scores as two dependent variables.

### Assessing Intelligence

To control the impact of general intelligence on brain structure (Colom et al., 2006; Jung and Haier, 2007; Takeuchi et al., 2011a), we measured participants’ general intelligence with the Combined Raven’s Test-Rural in China (revised by the Psychology Department of East China Normal University in 1994; Takeuchi et al., 2010, 2011b). This scale is adopted to test intelligence in a wide range for good reliability and validity (Wang, 2007). The Revised Combined Raven’s Test-Rural in China contains the Raven’s colored progressive matrix (A, B, and AB sets) and Raven’s standard progressive matrix (C, D, and E sets). This scale includes 72 non-verbal items. In every item, participants are asked to select the best answer from six or eight alternatives to fill a matrix with a missing piece.

### Image Data Acquisition and Preprocessing

Magnetic resonance imaging (MRI) data were acquired with a 3T scanner (Siemens Magnetom Trio TIM, Erlangen, Germany). A magnetization-prepared rapid gradient echo (MPRAGE) sequence was used to collect the high-resolution T1-weighted anatomical images (TR = 1900 ms; TE = 2.52 ms; TI = 900 ms; flip angle = 9; 176 slices; slice thickness = 1.0 mm; FOV = 250 mm × 250 mm; matrix size = 256 × 256).

Structural magnetic resonance images were processed with SPM8 software (Welcome Department of Cognitive Neurology, London, UK). Specifically, artifacts or anatomical abnormalities were first screened in SPM8. Subsequently, images were manually set along the anterior-posterior commissure (AC-PC) line to get better registration. Next, the new segmentation tool in SPM8 was employed to segment images into gray matter, white matter, and cerebrospinal fluid. The segmented images were submitted for registration, normalization, and modulation using SPM8 plus DARTEL (Ashburner, 2007). After that, Jacobian determinants were used to modulate images in order to conserve the absolute amount of gray matter (Good et al., 2002). Finally, the modulated gray matter images were smoothed with a 10 mm full-width at half-maximum (FWHM) Gaussian kernel to improve the signal-to-noise ratio.

### Region of Interest Extraction

We extracted six cortical ROIs from the Automated Anatomical Labeling atlas using the WFU PickAtlas Toolbox (Tzourio-Mazoyer et al., 2002). The ROIs (hippocampal, parahippocampal, lingual gyrus, precuneus, cuneus, IFG) were identical to those used in previous studies of structural correlation networks (Fink et al., 2009; Jung et al., 2010; Fink et al., 2014; Zhang et al., 2014; Boccia et al., 2015b; Jauk et al., 2015). These ROIs were resampled to the dimension of the voxel-based morphometry images. Subsequently, the ROI images were used as masks to extract the GMV within each ROI from the modulated, normalized gray-matter images using the REX toolbox (Massachusetts Institute of Technology, Cambridge, MA, USA). In order to eliminate the influence of non-interest covariates, subjects’ age, gender, and total brain volume (including gray matter, white matter, and cerebrospinal fluid) were submitted to a linear regression analysis for each ROI. The residuals of each regression were used for subsequent analysis to replace the raw ROI volume values (He et al., 2007; Bernhardt et al., 2011; Hosseini et al., 2012).

### Mediation Analysis

Before performing a mediation analysis, all data were normalized. The non-interest effect of age, gender, and general intelligence was removed from behavioral data (i.e., DT and Stroop-effect scores). We used Stroop-effect scores as the independent variable, DT as the dependent variable, and GMV of ROIs as mediators. To further investigate the effects of inhibition and GMV on facets of DT, we set up several models for originality and fluency, wherein originality and fluency of DT tasks were used as dependent variables.

**FIGURE 1:**
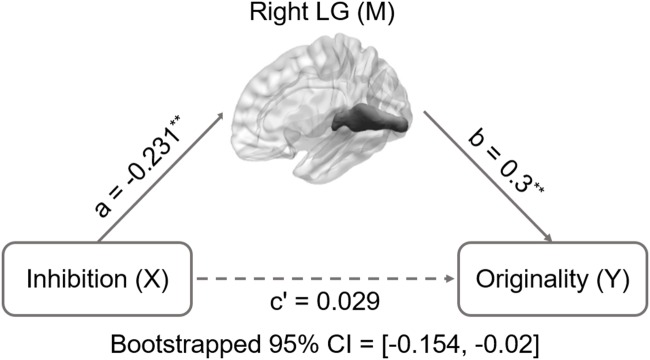
**Mediation analysis for right lingual gyrus volume and originality.** Path c’ is the direct effect of DT on inhibition functions after controlling for the GMV of lingual gyrus; the product of the paths a and b (ab) is the indirect effect of inhibition functions on originality through the GMV of right lingual gyrus. The GMV of lingual gyrus mediated the association between inhibition functions and originality (bootstrap estimate 95% confidence interval: -0.154, -0.02). ^∗∗^*p* < 0.001.

A simple mediation analysis was employed using SPSS macro with a 0.95 confidence level and 5000-bootstrap sample (Preacher and Hayes, 2004, 2008). In typical mediation analyses, several paths between the variables are estimated. The chief estimated path is the total effect of an independent variable X on a dependent variable Y (path c). This path comprises the direct effect of X on Y after controlling for mediator M (path c’) and the indirect effect of X on Y through M (i.e., path ab: product of path X → M and M → Y: **Figure 1**). After accounting for M, the mediation analysis was determined by assessing whether there is a significant distinction between the total (path c) and direct effects (path c’). When the zero is not encompassed within the confidence interval (CI), a significant difference between the indirect effect (path a b) and zero exists, which indicates that regional structural volume accounts for a statistically significant portion of the relationship between inhibition functions and DT.

## Results

### Increased ROIs Volume Linked to Decreased Inhibition Function and Increased DT (Originality and Fluency) Performance

All behavioral data were analyzed using SPSS 18.0 (SPSS Inc., Chicago, IL, USA). Partly confirming our first prediction, only lingual gyrus volume was positively associated with originality (*r* = 0.276, *p* < 0.01, corrected for multiple comparisons, shown in **Table 1**) and fluency (*r* = 0.269, *p* < 0.01, corrected for multiple comparisons, shown in **Table 1**), and negatively associated with inhibition function (*r* = -0.247, *p* < 0.05, corrected for multiple comparisons, shown in **Table 1**).

**Table 1:** Correlations between behavioral and volumetric measures.

			Region of interest		
				
Variable	Parahippocampal	Cuneus	Precuneus	Hippocampal	Lingual	Inferior frontal gyrus (IFG)
Inhibition	0.009	-0.182	-0.042	0.072	-0.247^∗^ (0.03)	-0.031
Originality	-0.022	0.101	0.023	0.050	0.276^∗∗^ (0.002)	0.001
Fluency	-0.028	0.072	-0.016	-0.014	0.269^∗∗^ (0.002)	-0.006


*^∗^*p* < 0.05 (two-tailed), ^∗∗^*p* < 0.001 (two-tailed), corrected for multiple comparisons. DT, divergent thinking. The values in the blackest represent the *P* value.*

**FIGURE 2:**
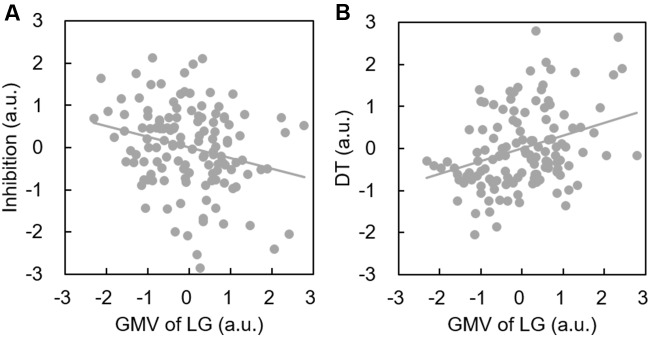
**Increased lingual gyrus volume is associated with better DT performance and poorer inhibition.** Scatterplots show a significant positive correlation between the GMV of the lingual gyrus and DT **(A)**; and a significant negative correlation between the GMV of the lingual gyrus and inhibition **(B)**.

Descriptive statistics are presented in **Table 2** and correlations between the behavioral variable and ROIs volume are presented in **Table 1**. Since only the relationship between behavioral data and lingual gyrus was significant (**Figure 1**), only lingual gyrus data were considered for further analysis (**Figure 2**).

**Table 2:** Descriptive statistics of subject demographics and psychological measures (*N* = 128; males = 49, females = 79).

Measure	*M*	*SD*	Range
Age	19.7	0.97	17–22
CRT	65.63	3.76	49–72
DT	77.06	28.76	24–176
Stroop	0.13	0.06	0.02–0.32


*CRT, Combined Raven’s Test; DT, divergent thinking.*

### Mediation Results

**FIGURE 3:**
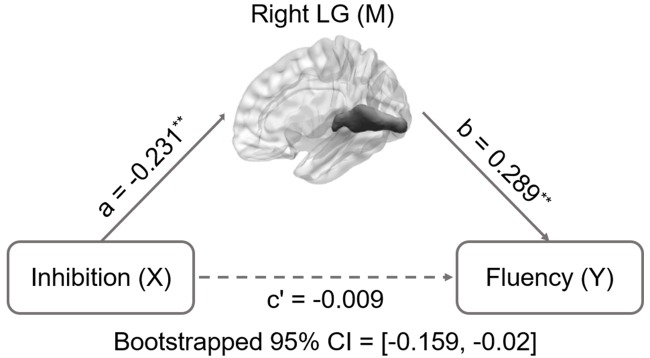
**Mediation analysis for right lingual gyrus volume and fluency.** Path c’ is the direct effect of DT on inhibition functions after controlling for the GMV of lingual gyrus; the product of the paths a and b (ab) is the indirect effect of inhibition functions through the GMV of right lingual gyrus on fluency. The GMV of lingual gyrus mediated the association between inhibition functions and fluency (bootstrap estimate 95% confidence interval [-0.159, -0.02]). ^∗∗^*p* < 0.001.

**FIGURE 4:**
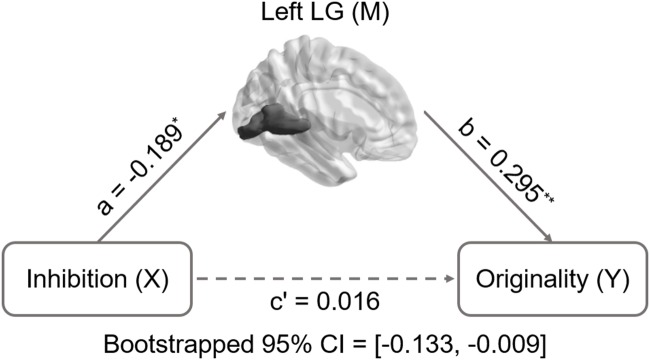
**Mediation analysis for the relationship between left lingual gyrus volume and originality.** Path c’ is the direct effect of DT on inhibition functions after controlling for the GMV of lingual gyrus; the product of the paths a and b (ab) is the indirect effect of inhibition function on originality through the GMV of left lingual gyrus. The GMV of lingual gyrus mediated the association between inhibition functions and originality (bootstrap estimate 95% confidence interval: -0.133, -0.009). ^∗^*p* < 0.05; ^∗∗^*p* < 0.001.

**FIGURE 5:**
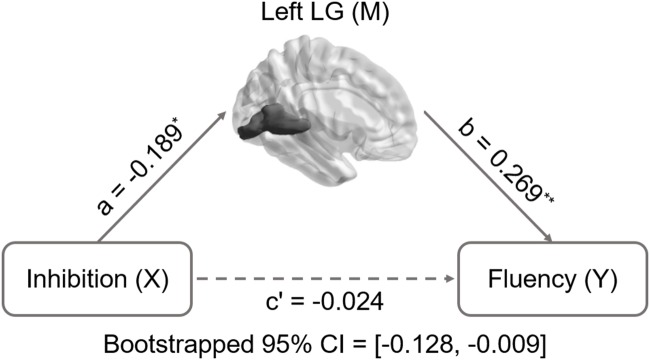
**Mediation analysis for left lingual gyrus volume and fluency.** Path c’ is the direct effect of DT on inhibition functions after controlling for the GMV of lingual gyrus; the product of the paths a and b (ab) is the indirect effect of inhibition functions through the GMV of left lingual gyrus on fluency. The GMV of lingual gyrus mediated the association between inhibition functions and fluency (bootstrap estimate 95% confidence interval [-0.128, -0.009]). ^∗^*p* < 0.05; ^∗∗^*p* < 0.001.

Inhibition was selected as the independent variable after controlling for intelligence, gender, and age. The right and left lingual gyrus were used separately as mediators to investigate a concrete neural basis for originality and fluency of DT. It was found that (1) the GMV of the right lingual gyrus accounts for a significant portion of the relationship between inhibition functions and originality (path a = -0.231, *p* = 0.005; path b = 0.3, *p* = 0.002; path a × b = -0.069, bootstrapped 95% CI = -0.154, -0.02; **Figure 2**); (2) GMV of the right lingual gyrus accounts for a significant portion of the relationship between inhibition functions and fluency (*a* = -0.231, *p* = 0.005; *b* = 0.289, *p* = 0.003; a × b = -0.067, bootstrapped 95% CI = -0.159, -0.02; **Figure 3**); (3) GMV of the left lingual gyrus accounts for a significant portion of the relationship between inhibition functions and originality (*a* = -0.189, *p* = 0.024; b = 0.295, *p* = 0.002; a × b = -0.056, bootstrapped 95% CI = -0.133, -0.009; **Figure 4**); and (4) GMV of the left lingual gyrus accounts for a significant portion of the relationship between inhibition functions and fluency (*a* = -0.189, *p* = 0.024; *b* = 0.269, *p* = 0.005; a × b = -0.051, bootstrapped 95% CI = -0.128, -0.009; **Figure 5**).

## Discussion

The present study used mediation analysis to classify the associations between GMV of classical regions located in TOP areas, inhibition function, and DT. The results showed two main findings: poorer inhibition function was associated with increased lingual gyrus volume, which in turn was associated with better DT performance; and the lingual gyrus GMV mediated the relationship between inhibition function and DT.

The association between lingual gyrus GMV and DT advances previous structural and functional neuroimaging evidence that suggests that greater lingual gyrus GMV volume is associated with better ideational fluency (Jauk et al., 2015) and activation of the left lingual gyrus is associated with better performance in processing of semantic relatedness (Zhang et al., 2014). Specifically, research consistently shows that the lingual gyrus is associated with visual memory (Bogousslavsky et al., 1987), vivid visual imagery (Belardinelli et al., 2009), and motion imagery (Malouin et al., 2003). The creative process necessitates the generation of new visual imagery (Anderson and Helstrup, 1993; LeBoutillier and Marks, 2003). Moreover, visual imagery is an important strategy for DT (Gilhooly et al., 2007; Fink et al., 2014). As suggested by Guilford (1967), the prefrontal cortex draws task-relevant information from long-term memory stored in the TOP areas and temporarily represents it in the working memory space. This allows the prefrontal cortex cognitive flexibility when combining the retrieved information to form new combinations. Lingual gyrus GMV may be one important neural marker of long-term memory (specifically visual memory) storage.

The current findings demonstrate a negative association between lingual gyrus volume and inhibition function. Although there is little experimental evidence for the direct relationship between inhibition function and lingual gyrus, Dietrich (2004) proposed that inhibition function would modulate the associative activity among knowledge nodes in TOP regions. Furthermore, some researchers (Radel et al., 2015) suggest that the inhibition function may automatically modulate the spreading of concept activation. Impaired inhibition function would activate a greater number of concepts in working memory, with several weak links to the initial concept. Further, the working memory buffer – with its ability to sustain online processing in real time – may be a prerequisite access to long-term memory (Dietrich, 2004). Importantly, it is widely accepted that TOP regions is the site of long-term memory storage (Gilbert, 2001; Dietrich, 2004). Therefore, the negative association between inhibition function and lingual gyrus volume found in our study may indicate that poorer inhibition function is associated with better visual memory storage, and thus, greater GMV in TOP regions. However, according to Mechelli et al. (2000), they used positron emission tomography to investigate the effect word length and visual contrast in the visual cortex during reading. Results indicate that the effect of lingual gyrus enhanced by low-contrast words reflects global shape processing when reading. In other words, the GMV in lingual gyrus may be related to the global shape processing ability in incompatible trials in Stroop task instead of the amount of memory storage. But task-related brain activity is different from VBM. Task-related brain activity is the correlation between task phase and brain activity, which might be a kind of momentary effect, while VBM provide structural information, which may be a kind of accumulative effect. So it is not obvious that the GMV of lingual gyrus is associated with inhibition interference in the Stroop task.

Inconsistent with previous studies showing that inhibition function is positively or negatively associated with creative cognition, we find that inhibition function is not directly associated with DT. Creative thought has been variably associated with focused attention and effective inhibition control (Beaty et al., 2014b, 2016), disinhibition and defocused attention (Radel et al., 2015), or a flexible adaption of inhibition control (Cassotti et al., 2016a). While some researchers suggested that inhibitory control hindered the creative potential (Radel et al., 2015), however, other studies reported that generating creative solutions to a problem require the inhibition of past inappropriate ideas that induce fixation phenomena (Cassotti et al., 2016a,b). In addition, many famous scientists indicated when original thoughts enter into their brain they are not deliberately aimed at solving the problem they were trying to solve. The possible interpretation is that creative cognition involves a flexible adaptation of inhibition function, as a line of studies that creative cognition may involve a two-stage process that include idea generation and idea evaluation (Finke et al., 1992). Researchers often think idea generation as a bottom-up process which is associated with diffuse attention. By contrast, idea evaluation is regarded as a top-down process with focused attention and cognitive control (Jung et al., 2013). In the idea generation stage, diffuse attention and disinhibition may contribute to ideas generation, as concepts used for generating uncommon ideas are dispersedly stored in semantic memory (Tyler and Moss, 2001; Thompson-Schill et al., 2006). Therefore, in this stage, it may show a negative correlation pattern between inhibition and creativity. While in the idea evaluation stage, inhibition control plays a key role to screen creative ideas/associations by suppressing prepotent and common ideas. Thus, a positive correlation pattern between inhibition and creativity would occur. In the present study, the results indicated that inhibition is negatively associated with DT in an indirect way. Inhibition function is negatively associated with the GMV of lingual gyrus–a brain region supporting for visual memory (Bogousslavsky et al., 1987), vivid visual imagery (Belardinelli et al., 2009), and motion imagery (Malouin et al., 2003). And then, the GMV of lingual gyrus is positively associated with DT. This may conform to the suggestion that diffuse attention and disinhibition contribute to ideas generation by using concepts dispersedly stored in semantic memory (Tyler and Moss, 2001; Thompson-Schill et al., 2006). Importantly, other uncontrolled factors such as emotion, motivation, and personality traits may influence the relationship between inhibition function and DT due to the complexity of creative cognition. In the future, more factors should be taken into consideration to examine the relationship between the inhibition function and DT. In addition, inconsistent results obtained across previous studies may be partly because various ways to define and assess inhibition. How to define inhibition ranges from cognition inhibition to motor inhibition (Harnishfeger, 1995; Aron, 2007). Furthermore, experimental paradigms for assessing inhibition includes semantic inhibition of return task, intentional forgetting task, retrieval-practice paradigm, flanker task, stop-signal task, Stroop task, and so on, which are used to different kinds of inhibition. In the future, it is necessary to thoroughly examine the relationship between inhibition and creativity cognition.

The present study also found that the GMV of the lingual gyrus mediates the relationship between inhibition function and DT. Although previous researches (Benedek et al., 2012b; Jauk et al., 2015; Radel et al., 2015) in behavioral and cognitive neuroscience indicate that inhibition influences DT, and the volume of posterior brain areas are related to DT, few have simultaneously examined the relationships between inhibition, brain structure, and DT. Results indicate that lingual gyrus volume mediated the relationship between inhibition function and DT. Poorer inhibition function may have led to a greater amount of visual information or imagery stored in the lingual gyrus. Demand for large information storage contributes to the increased lingual gyrus volume. Visual information from the lingual gyrus is drawn to the working memory space and the retrieved information is combined to form new combinations. That is, more information helps form new combinations and may explain the inhibition-lingual gyrus-DT pathway. However, because the mediation models tested in the present study did not identify the direction of the influence between inhibition and DT, longitudinal studies are needed to clarify this issue.

In the present study, only the volume of the lingual gyrus was found to mediate the relationship between Inhibition function and DT, compared to the volume of hippocampal, parahippocampal, precuneus, and cuneus regions. This may be because the lingual gyrus is associated with visual memory (Bogousslavsky et al., 1987) and vivid visual imagery (Belardinelli et al., 2009). Further, the product improvement tasks used in the present study required participants to list ways in which a product could be changed to be more interesting or unusual. Visual imagery may play an important role in this process. Therefore, lingual gyrus volume may mediate the relationship between inhibition function and product-improvement performance. Future research should examine possible relationships between the performance in each verbal activity on The Torrance Tests of Creative Thinking (Asking, Guessing Causes, Guessing Consequences, Product Improvement, Unusual Uses of Cardboard Boxes, and Just Suppose) and other kinds of memories to clarify this issue (Krumm et al., 2014a).

We found that the volume of IFG is not related with inhibition function and DT. Although, these results are inconsistent with previous functional imaging studies which have repeatedly demonstrated inhibition control cortex plays a crucial role in the creative thinking (Fink et al., 2009, 2015; Abraham et al., 2012b; Beaty et al., 2014a; Mayseless et al., 2015). There are a line of psychology and psychiatry studies showing that several mental states associated with impaired executive functioning, including ADHD (Healey and Rucklidge, 2006; White and Shah, 2006), fronto-executive disfunctioning/schizophrenia (Keri, 2009), lateral frontal lesions (Chrysikou et al., 2013), and cannabis intoxication (Morgan et al., 2010) can lead to creative performance. Obviously, the contradiction exists across abovementioned studies. Therefore, some researchers in the creative field speculated that creative cognition may involve a two-stage process including idea generation and idea evaluation (Finke et al., 1992). These researchers commonly think idea generation as a bottom-up process which is associated with diffuse attention. By contrast, idea evaluation is regarded as an top-down process with focused attention and cognitive control (Jung et al., 2013). Furthermore, it has been suggested by a model that idea generation and idea evaluation are separately depend on the default and the control networks (Jung et al., 2013). In the present study, we find that the GMV of IFG is not significantly related with DT, but the GMV of lingual gyrus is related with DT. IFG is classically associated with inhibitory control (Aron et al., 2004, 2014; Houdé et al., 2010). Lingual gyrus is a brain region supporting visual memory (Bogousslavsky et al., 1987) and vivid visual imagery (Belardinelli et al., 2009), which is a region important for idea generation. In other words, idea generation and idea evaluation (also the default and the control networks) may show little cooperation in this DT task. These results are consistent with the EEG studies indicating that successful performance at ideational fluency tasks do not depend on cortical arousal in prefrontal regions (Martindale and Hasenfus, 1978; Mölle et al., 1999). Moreover, individuals with PFC damage solve insight problems better than the healthy counterparts (Reverberi et al., 2005). Importantly, a fMRI study found that Uncommon-Use-task reliably activated regions of occipito-temporal cortex without regions of lateral prefrontal cortex compared with the Common-Use-task (Chrysikou and Thompson-Schill, 2011). In addition, previous studies usually found IFG is activated when individuals are performing a creativity task. Task-related brain activity is the correlation between task phase and brain activity, which might be a kind of momentary effect, while VBM provide structural information, which may be a kind of accumulative effect. Although previous studies indicate that the prefrontal cortex is associated with inhibition function, at the same time, prefrontal cortex is activated during idea generation process, it is not obvious that the GMV of IFG is associated with inhibition function and DT.

However, there are some limitations in the present study. First, it is not sufficient to attribute the link between inhibition and DT to memory processes due to a lack of working memory and long-term memory assessment. In future research, memory measurements should be involved to provide a substantial evidence to examine the mechanism underlying the association between inhibition and lingual gyrus. Second, the Stroop task used in this study may be not an appropriate measurement to test inhibition function, as this version of Stroop task lacks a neural condition. Because of this, we are not sure the difference is due to inhibition function or attention control. Modified task is required to test the relationship between inhibition function and DT in future research.

In summary, the present findings – lingual gyrus volume mediated the relationship between inhibition function and DT – are important because they potentially explain the influence of inhibition on DT through the posterior cortex. Limited research has simultaneously examined inhibition, brain structure, and DT behavior to identify their interrelationships and the mechanisms through which inhibition influences DT. The results provide novel evidence for the role of inhibition function in creative thinking by its influence on the amount of information stored in long-term memory. However, longitudinal studies are needed to establish the long-term impact of lingual gyrus volume and the direction of influence in our model.

## Author Contributions

LZ, LQ, QC, JQ, and DY programmed the experiments and analyzed the data; LZ and WY drafted the manuscript and LZ, LQ, QC, WY, MX, and XY provided critical revisions; LQ prepared the figures.

## Conflict of Interest Statement

The authors declare that the research was conducted in the absence of any commercial or financial relationships that could be construed as a potential conflict of interest.

## Abbreviations

DT: divergent thinking
GMV: gray matter volume
ROIs: regions of interest
TOP: temporal, occipital and parietal
VBM: voxel based morphometry
MRI: magnetic resonance imaging
